# Heterogeneity of Response and Immune System Activity during Treatment with Nivolumab in Hepatocellular Carcinoma: Results from a Single-Institution Retrospective Analysis

**DOI:** 10.3390/cancers13020213

**Published:** 2021-01-08

**Authors:** Ilario Giovanni Rapposelli, Serena De Matteis, Paola Lanuti, Martina Valgiusti, Giulia Bartolini, Paola Ulivi, Giorgia Marisi, Federica Pedica, Valentina Burgio, Giovanni Luca Frassineti, Stefano Cascinu, Andrea Casadei-Gardini

**Affiliations:** 1 Department of Medical Oncology, IRCCS Istituto Romagnolo per lo Studio dei Tumori “Dino Amadori”—IRST, 47014 Meldola, Italy; martina.valgiusti@irst.emr.it (M.V.); giulia.bartolini@irst.emr.it (G.B.); luca.frassineti@irst.emr.it (G.L.F.); 2 Department of Experimental, Diagnostic and Specialty Medicine, University of Bologna, 40126 Bologna, Italy; serenadema85@gmail.com; 3 Department of Medicine and Aging Sciences, University “G. d’Annunzio”, Chieti-Pescara, 66100 Chieti, Italy; p.lanuti@unich.it; 4 Center for Advanced Studies and Technology (C.A.S.T.), University “G. d’Annunzio”, Chieti-Pescara, 66100 Chieti, Italy; 5 Biosciences Laboratory, IRCCS Istituto Romagnolo per lo Studio dei Tumori “Dino Amadori”—IRST, 47014 Meldola, Italy; paola.ulivi@irst.emr.it (P.U.); giorgia.marisi@irst.emr.it (G.M.); 6 Department of Pathology, IRCCS San Raffaele Scientific Institute, 20132 Milan, Italy; pedica.federica@hsr.it; 7 Unit of Oncology, IRCCS San Raffaele Scientific Institute, 20132 Milan, Italy; burgio.valentina@hsr.it (V.B.); cascinu.stefano@hsr.it (S.C.); casadeigardini@gmail.com (A.C.-G.); 8 School of Medicine, Vita-Salute San Raffaele University, 20132 Milan, Italy

**Keywords:** hepatocellular carcinoma, immunotherapy, pattern of response, peripheral blood mononuclear cells

## Abstract

**Simple Summary:**

Immunotherapy is an emerging treatment in hepatocellular carcinoma, both alone and in combination. The advent of this new approach raises challenges for the interpretation of response assessment due to the peculiar patterns of mixed responses, pseudoprogression and hyperprogression. Furthermore, there are no criteria to drive selection of treatment strategy. We analyzed data from the first 10 patients treated with nivolumab in our institution and we identified different patterns of response according to the lesion’s site. Furthermore, we analyzed blood samples from the first four patients, and found differences, between a patient with shorter survival and the other three, that may provide insight into mechanisms underlying the different activities of nivolumab. Although we analyzed data from a small number of patients, our results can help to understand mechanisms of immunotherapy activity in order to define the most appropriate treatment strategy for each patient.

**Abstract:**

Treatment of hepatocellular carcinoma (HCC) is rapidly evolving, with many new therapeutic options; in particular, immunotherapy (IT) is acquiring a major role, even in combination regimens. Despite these promising results, an important limitation is the lack of prognostic and predictive factors that prevent provision of a tool for patient stratification in order to select the most appropriate strategy. Furthermore, response assessment can be challenging with IT due to peculiar patterns such as mixed responses or pseudoprogression. We analyzed biological and clinical features from the first 10 HCC patients treated with nivolumab in our institution. Analysis of patterns of response in CT assessment revealed complete response in pulmonary lesions, along with heterogeneous behavior in the liver and other organ lesions. Peripheral blood mononuclear cells (PBMC) analysis in the first four patients showed unique alterations in a patient with poor prognosis, both at baseline (lower percentage of effector T cells, higher percentage of natural killer T [NK/T] cells) and during treatment with nivolumab (decrease in nonclassical monocytes, increase in monocytic myeloid-derived suppressor cells [MO-MDSC]), suggesting a possible prognostic role for these features. Although obtained in a small cohort of patients, our results open a new perspective for understanding mechanisms underlying IT outcomes in HCC patients.

## 1. Introduction

Hepatocellular carcinoma (HCC) accounts for 75–85% of cases of liver cancer, which is the fourth leading cause of cancer death worldwide [1]. HCC usually arises in the context of a preexisting liver disease (i.e., cirrhosis), mostly as a consequence of chronic hepatitis B virus (HBV) or hepatitis C virus (HCV) infection, alcohol abuse or nonalcoholic fatty liver disease [2]. Most cases are diagnosed at an advanced stage, only amenable to systemic therapy.

Although for a long time the tyrosine kinase inhibitor (TKI) sorafenib was the only available systemic therapy for advanced HCC [3], we are currently seeing an evolving landscape, with many other TKI options both in first-line (lenvatinib) [4] and second-line settings (regorafenib, ramucirumab and cabozantinib) [5,6,7,8]. Furthermore, immunotherapy (IT) has an emerging role; besides the promising results from phase 2 trials with nivolumab and pembrolizumab, two monoclonal antibodies (mAb) against the programmed cell death protein 1 (PD-1) [9,10], most recent developments include combination strategies such as nivolumab plus ipilimumab (mAb anti-Cytotoxic T-Lymphocyte Antigen 4) [11], TKI plus IT (e.g., lenvatinib + pembrolizumab) [12] and the association of atezolizumab and bevacizumab, two mAbs against programmed death-ligand 1 (PD-L1) and vascular endothelial growth factor (VEGF), respectively [13]. The latter regimen improved both overall survival (OS) and progression-free survival (PFS) compared with sorafenib in a phase 3 trial [13].

The increasing availability of new options for systemic therapy of HCC (IT, TKI and combinations) raises the need for prognostic and predictive factors in order to stratify patients and choose the most appropriate treatment strategy.

Another challenge raised by the increasing use of IT is the assessment of response, leading to the establishment of specific criteria (immune Response Evaluation Criteria in solid Tumors, iRECIST) [14]. In this context, the possible divergent behavior of lesions from different organs is even more challenging with IT than chemotherapy, since it may derive not only from tumor heterogeneity and different treatment efficacy, but also from other phenomena that must be recognized and properly assessed (e.g., pseudoprogression). This is crucial to optimize treatment strategy, e.g., combining systemic and locoregional therapies (transarterial chemoembolization, transarterial radioembolization, surgery or radiofrequency) for nonresponding lesions.

In this work, we integrated clinical and biological features obtained from the first 10 patients treated with nivolumab in our institution in order to identify possible prognostic factors and to analyze patterns of response. We retrieved CT scans for measurement of lesions and collected results from laboratory analysis of blood samples; given the peculiar mechanism of action of IT, we also analyzed blood samples from the first four patients in order to assess baseline and on-treatment levels of peripheral blood mononuclear cells (PBMC).

## 2. Results

### 2.1. Treatment Outcomes and Pattern of Response Analysis

The main characteristics of the patients enrolled in the study are summarized in Table 1. Median age at first dose of nivolumab was 66 years. All but one of the patients received locoregional treatment. All patients previously received sorafenib; only one patient underwent sorafenib suspension for toxicity, all others for progressive disease (PD).

**Figure 1:**
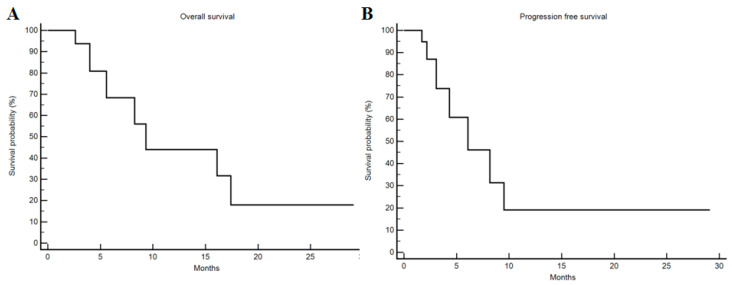
Kaplan–Meier analysis of overall survival and progression-free survival. (**A**) Median overall survival was 9.3 months (95% CI 2.6–17.4). (**B**) Median progression-free survival was 6.1 months (95% CI 1.7–9.5).

Median OS was 9.3 months (95% CI 2.6–17.4) and median PFS was 6.1 months (95% CI 1.7–9.5; Figure 1). The six-month OS rate was 70%, the nine-month OS rate was 60% and the one-year OS rate was 50%.

For basal characteristics, the univariate analysis identified eosinophil count (<50 versus >50; *p* value = 0.0009), increase in neutrophils (*p* = 0.0388) and alpha-fetoprotein (*p* = 0.0363) continuous variables as potential prognostic factors for poorer OS. No other positive correlations were found.

Disease control rate (DCR) in our population was 70% (one partial response and six stable disease), while three patients experienced PD at first response assessment (Table 2). Three patients are still on treatment (23.6 months, 29.1 months and 15.5 months from starting nivolumab), six suspended nivolumab because of PD and one had to withdraw due to an intercurrent illness (COVID-19) and subsequently experienced PD.

Six patients were eligible for pattern of response analysis, three were excluded since imaging was not available for revision and lesion measurement, and one patient had no radiological response assessment during treatment but was evaluated on clinical progression.

**Figure 2:**
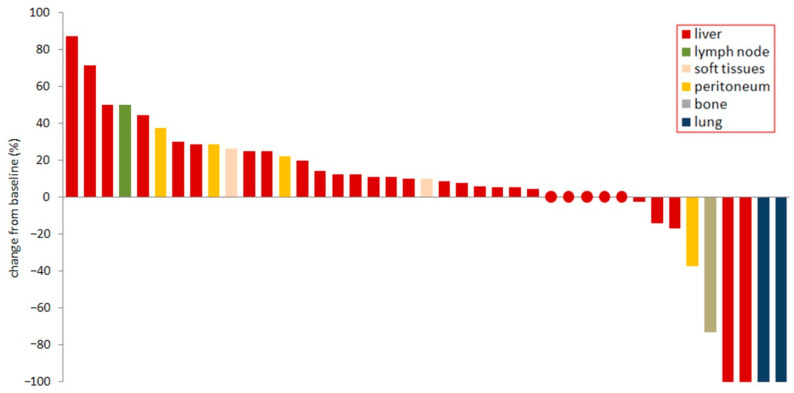
Best percentage change in single lesions. Best percentage change from baseline depicted for every single lesion analyzed. Circles indicate liver lesions with null change.

In a revision of CT scans from eligible patients, we found a total of 41 lesions suitable for analysis (31 liver, 4 peritoneum, 2 lung, 2 soft tissues, 1 lymph node and 1 bone). Analyzing best response profiles for single lesions according to iRECIST criteria [14], we found that liver lesions showed heterogeneous behavior (2 CR, 20 SD, 9 PD; 6%, 65%, 29%, respectively), all pulmonary lesions had a complete response (100%), and lesions in other organs also showed a heterogeneous response (2 PR, 1 SD, 5 PD; 25%, 12,5%, 62,5%, respectively; Figure 2).

**Figure 3:**
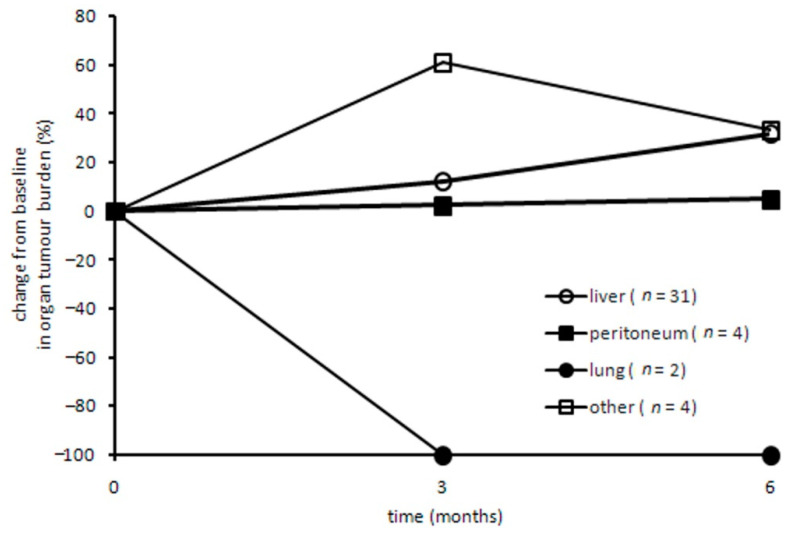
Change in global tumor burden for each organ. All lesions analyzed were organized by organ (liver, peritoneum, lung and other) and the percentage change from baseline was reported for every organ.

Then, we analyzed the global tumor burden for each organ (liver, lung, peritoneum and other), pulling together data from all patients. Data from the first three CT assessments were evaluated, since some patients interrupted treatment after this time point due to PD. This analysis, besides complete response for pulmonary lesions at first evaluation during treatment, globally showed slow increase in liver lesions (+32%), stability of peritoneal nodules (+5%) and faster progression in other organs (+33%; Figure 3). The initial peak in the “other organs” graph resulted from pseudoprogression for a single bone lesion.

Grade 3/4 treatment-related adverse events occurred in 1 out of 10 patients. Treatment-related adverse events were two immune-mediated pneumonia (grade 3 and grade 1), one hypothyroidism (grade 2) and rash in one patient (grade 2).

### 2.2. Evaluation of Immune Cell Subsets

Multicolor flow cytometry was performed on fresh PBMC obtained from the first four patients enrolled in this study at baseline and after 14 and 28 days of treatment with nivolumab to monitor immune system evolution (Figure S1).

**Figure 4:**
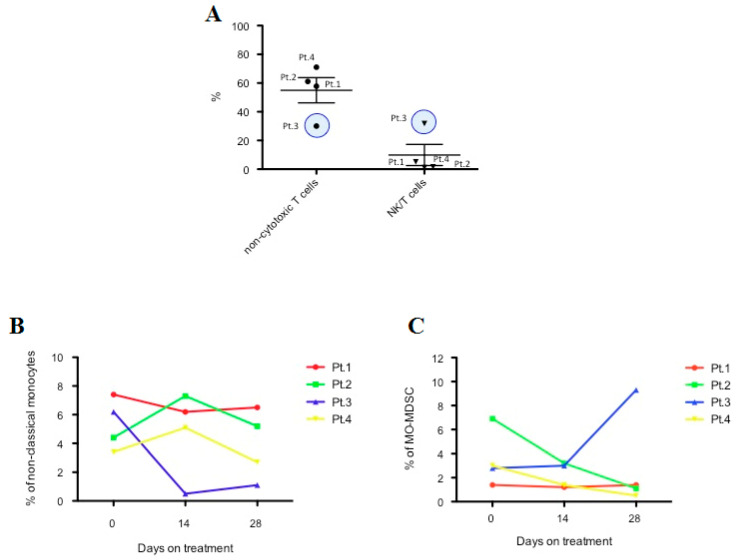
Analysis of immune cell subsets by flow cytometry. (**A**) Percentage of noncytotoxic T cells and NK/T cells at baseline. (**B**) Percentage of nonclassical monocytes over the course of therapy with nivolumab (at baseline, after 14 and after 28 days). (**C**) Percentage of monocytic myeloid-derived suppressor cells (MO-MDSC) over the course of therapy with nivolumab (at baseline, after 14 and after 28 days). Analysis was performed in the first four patients. Numbering of patients in the three panels is the same as reported in Table 2.

At baseline, we observed remarkable differences between a patient with a poor prognosis (Pt.3; same numbering as in Table 2) and the other three patients (Pt.1, Pt.2 and Pt.4). Specifically, Pt.3 showed a lower percentage of circulating noncytotoxic T cells and a higher percentage of natural killer T (NK/T) cells than the other patients (Figure 4A). During the course of treatment, we reported variations in the percentages of classical and intermediate monocytes after only 14 days of treatment (Figure S2). Interestingly, the increase in classical monocytes observed in Pt.3 after 14 days of treatment was consistent with reports of standard blood count examination, where it was confirmed also in another patient with a short time to progression (Figure S3).

In addition, as reported in Figure 4B,C, Pt.3 showed a decrease in nonclassical monocytes over the course of therapy and a time-dependent increase in monocytic myeloid-derived suppressor cells (MO-MDSC), which was different from the other three patients.

## 3. Discussion

This study highlights several points that could provide new insight for IT in HCC. First of all, the study remarks upon the different pattern of response based on the lesion’s site. Our data showed a complete response in two pulmonary lesions and a heterogeneous response of hepatic and other organ lesions (which showed an overall stability). The different patterns of response of lung versus other lesions might be important to understand the mechanism of response to IT, which is influenced by the tumor microenvironment (TME).

Dissociated responses are not uncommon with IT, or in cancers other than HCC [15]. More specifically, organ-specific, heterogeneous behavior of HCC lesions was previously described, with a recent work reporting a different response (disease control vs PD) between hepatic and extrahepatic lesions in 16 out of 39 patients analyzed (41%), with hepatic lesions being less responsive and lung metastases the most responsive [16]. Furthermore, a better response of lung lesions was previously reported in other cancers treated with IT [17,18]. On the other hand, the presence of liver metastases was also linked to a reduced response rate and a worse prognosis (shortened PFS) in melanoma and non-small cell lung cancer (NSCLC) [19,20].

The existence of mixed responses is a well-known feature also in patients treated with chemotherapy or targeted therapy, and is related to tumor heterogeneity [21]. In patients treated with IT, given the peculiar mechanism of action, mixed responses depend rather on different TME, i.e., a milieu of cancer cells, immune cells, blood vessels, extracellular matrix and signaling molecules that can influence sensitivity to IT [22]. Although different lymphocyte infiltration and distribution, resulting in the so-called inflamed, immune-excluded and immune desert phenotypes [22], was previously linked to a different sensitivity to IT, the picture is even more complex, and has to take into account a plethora of other actors.

A difference in TME between primary and metastatic lesions was previously described in several cancers [23,24,25], and a TME heterogeneity in metastases with a different behavior was reported in ovarian cancer [26].

HCC lesions are typically immunogenic since they express shared tumor antigens and private neoantigens arising from gene mutations [27]. Moreover, they contain tumor-infiltrating lymphocytes expressing PD-1, and PD-L1 expression is not significantly different between primary and metastatic lesions, making HCC a good candidate for treatment with PD-1 inhibitors, such as nivolumab [9,28]. Despite these features, the antitumor immune responses in HCC are subverted by the action of stromal cells and immunoinhibitory molecules [27]; indeed, the liver microenvironment, both in normal biology and in disease, is a complex interplay between immunity and tolerance [29]. Immune tolerance is essential in normal physiology, since the liver deals with a huge antigen load from the gastrointestinal tract, including nonpathogenic gut commensals that do not require an inflammatory response [29]. Immune tolerance is mostly mediated by T regulatory cells (Tregs) through interaction with (and activation by) other cell types, such as Kupffer cells, liver sinusoidal endothelial cells, hepatic stellate cells and resident dendritic cells, with several mediators involved in this process, such as interleukin 10 (IL-10), transforming growth factor-β (TGF-β) and PD-L1 [30]. For example, Kupffer cells induce tolerance by expression of IL-10, which induces Tregs and PD-L1 [29]. On the other hand, HCC mostly arises in the context of cirrhosis, which sees a chronic inflammatory state resulting in fibrosis through a persistent, abnormal “wound healing-like” process that overtakes immune-mediated cancer surveillance [27]. In this context, immune inhibition and permanent exposure to tumor antigens result in T-cell exhaustion [27], with poor effector function and sustained expression of inhibitory receptors [31]. This comes together with systemic immune impairment in cirrhotic patients [29].

Immune dysfunction is finally a hallmark of HCC, i.e., the tissue contains unique populations of T cells with functional impairment [32]. Indeed, HCC lesions show an enrichment in Tregs, while CD8^+^ T cells are restricted to borders of tumors [27]; moreover, CD8^+^ T cells in HCC are dysfunctional, and both CD4 and CD8 lymphocytes have an increased expression of the inhibitory receptor TIM-3 and are replicative senescent [33]. Not surprisingly, the efficacy of T-cell responses is limited in HCC [34]. Furthermore, the TME of HCC stimulates polarization of macrophages toward the phenotype of tumor-associated macrophages, thereby supporting tumor progression with their immunosuppressive functions, in addition to promoting tumor cell invasion, angiogenesis and metastatic process [27]. Finally, HCC cells produce a large number of immunosuppressive molecules such as TGF-β and IL-10 [27].

Further confirming the immunosuppressive features of the liver microenvironment, liver metastases, in patients affected by melanoma and NSCLC, showed reduced CD8^+^ T-cell infiltration [19].

Another important aspect of this study is the higher response observed in small-sized lung lesions (<1 cm), consistent with other results obtained in other cancers treated with IT [35]. We also reported a case of pseudoprogression for a single bone lesion (Figure 3), that did not affect the global result for the “other organs” subset (PD). Pseudoprogression is a well-known phenomenon in cancer IT, observed in about 2–10% of patients treated with immune checkpoint inhibitors, and ascribed to an increase inexisting lesions and/or the appearance of new ones due to infiltration by activated T cells before response [36]; it was also previously described in HCC [37].

We also reported a case of hyperprogression (HPD), a possible outcome of treatment with IT also described in HCC. A recent work highlighted a 12.7% prevalence of HPD in HCC patients treated with nivolumab, while the phenomenon did not occur during therapy with the antiangiogenic TKI regorafenib [38]. Among the risk factors associated with HPD, elevated neutrophil-to-lymphocyte ratio (NLR) and certain polymorphisms of *VEGFR2* (rs1870377 A/T and A/A) were reported [38,39]. It is well known that an elevated NLR reflects changes in TME that favor disease progression [40]; on the other hand, VEGF has several immunosuppressive activities, such as Tregs induction, immunosuppressive cytokine release and myeloid-derived suppressor cells (MDSC) activation [41]. As a straight consequence, antiangiogenic drugs may compensate for limitations of IT [38], and many recent clinical trials were designed to exploit this combination [12,13].

Our cytometric analysis highlighted some immune system features in a small cohort of patients, suggesting a prognostic role for some cell subpopulations. Results observed in a patient with poor prognosis (Pt.3: lower percentage of non-cytotoxic T cells, higher of NK/T cells) were consistent with previous reports; indeed, an elevated CD4/CD8 ratio is predictive of recurrence-free survival [29], while overstimulation of NK/T cells, a subpopulation lying at the interface between the innate and adaptive immune system, can induce anergy and determine a switch toward an immunosuppressive phenotype, facilitating tumor progression and immune escape [42]. Furthermore, the presence of a T cell subpopulation (FOXP3^+^/CD3^+^/CD4^+^/CD56^+^) with a regulatory phenotype, induced by TGF-β, is well known in HCC tissue, and their presence is inversely correlated with patient survival [43]. FOXP3^+^ Treg cells are also increased in peripheral blood from HCC patients [27].

Interestingly, other cell subsets that emerged from our analysis were the nonclassical monocytes and MO-MDSC. In Pt.3, with a shorter survival, we observed during treatment a decrease in nonclassical monocytes, which contribute to cancer immunosurveillance through NK cell recruitment and activation [44] and are the primary producers of the inflammatory cytokines IL-1β and tumor necrosis factor-α (TNF-α) [45]. On the other hand, the increase in MO-MDSC in Pt.3 was consistent with previous reports showing an increase, both in blood and in tumor tissue of HCC patients, of a population of MDSC with immunosuppressive activity through the induction of Tregs [46] and the regulation of hepatic NK-cell activity via TGF-β [47]. Furthermore, the amount of MDSC was linked to serum concentrations of IL-10 (that has immunosuppressive activity) and to tumor progression and poor prognosis [48].

For basal characteristics, this study highlighted eosinophil count, increase in neutrophils and alpha-fetoprotein as potential prognostic factors for overall survival. In a previous study, Orsi et al. demonstrated a negative prognostic impact of low eosinophil count in a training cohort and in two validation cohorts in patients treated with sorafenib [49]. Our study reports, for the first time, the prognostic role of this peripheral cells in advanced HCC patients treated with IT. The prognostic role of eosinophils in patients treated with IT was already described in other neoplasms [50,51,52]. Evidence exists that the TME can be regulated by eosinophils and neutrophils [52,53], possibly underlying their prognostic role in patients treated with IT. The role of these peripheral cells should be further investigated; conversely, the prognostic role of alpha-fetoprotein in advanced HCC patients was well described in several studies [54].

Finally, median OS of our study was lower compared to the trial by El-Khoueiry et al., but DCR was similar [9]. Compared to this trial, patients with a Child–Pugh score of six or more were more numerous in our series (60% vs. 31%). Furthermore, while all patients treated in our series previously received sorafenib, and 9 out of 10 underwent suspension of the TKI because of disease progression, in the above trial there was a fraction of patients (26%) that did not previously receive systemic therapy and, even more remarkably, 32% of patients were not treated with sorafenib. Indeed, the line of therapy (first vs. later) was shown to be a predictor of OS for nivolumab in a univariate analysis of 233 patients from a real-world setting [55]. These differences may account, at least partially, for the lower survival outcomes reported here.

This study has some limitations. First of all, we analyzed a small sample, particularly regarding PBMC analysis. Furthermore, due to the retrospective nature of the study, we analyzed only two pulmonary lesions, since CT scans from other patients with lung lesions were not available for image review.

In conclusion, our results provide a new perspective for new strategies in the IT era for HCC patients. The different organ sensitivity to nivolumab (and other immunotherapeutic agents) could lay the ground for improvement of treatment strategy, i.e., local treatment (e.g., surgery or radiation therapy) for growing lesions, while keeping the same systemic therapy [56,57]. This is especially relevant in HCC, given the immunosuppressive microenvironment typically associated with liver lesions and the availability of other locoregional therapies (embolization, ablation) that, along with local disease control, could induce a release of tumor antigens and result in enhancement of IT activity [27,29].

The preliminary results obtained from PBMC analysis, and in particular the findings in the patient with poor prognosis, shed light on the mechanisms underlying HPD, a possible outcome of treatment with IT that is not uncommon in HCC [38].

Given the small sample size and the retrospective nature of the present work, these results need further confirmation, possibly in a prospective setting.

## 4. Materials and Methods

### 4.1. Patients and Treatment

The study population derived from retrospectively collected data of patients treated with nivolumab for advanced-stage (BCLC-C) or intermediate-stage (BCLC-B) HCC, deemed not eligible for first- or for retreatment with surgical or locoregional therapies. The overall cohort included only Western patients that started treatment between April 2018 and March 2020. All patients were treated at the IRCCS Istituto Romagnolo per lo Studio dei Tumori “Dino Amadori”—IRST (formerly Istituto Scientifico Romagnolo per lo Studio e la Cura dei Tumori—IRST—IRCCS; Meldola, Italy). Eligible patients had HCC diagnosis confirmed histologically or confirmed clinically in accordance with international guidelines. Patients were required to have at least one untreated target lesion that could be measured in one dimension, according to the modified Response Evaluation Criteria in Solid Tumors (mRECIST) [58].

For each patient, treatment with nivolumab (off-label for HCC in Italy) was requested on an individual basis and approved by institutional and national regulatory committees. All patients signed informed consent before treatment.

All patients received nivolumab 3 mg/kg. Information on blood tests carried out at baseline (the day before the start of treatment) was collected.

Adverse events were graded using the National Cancer Institute Common Terminology Criteria for Adverse Events (NCI-CTCAE) version 4.03 and seriousness of adverse events was recorded.

Treatment continued until the occurrence of radiologic progression, as defined by iRECIST, symptomatic progression, unacceptable adverse events or death.

Tumor response was evaluated every three months with contrast-enhanced computed tomography (CT) with triphasic scanning technique. Tumor response was evaluated by modified Response Evaluation Criteria in Solid Tumors (mRECIST) [58] and immune Response Evaluation Criteria in solid Tumors (iRECIST) [14].

The present study was approved by local ethics committee and informed consent was obtained from each patient for use of biological material for research purposes (CEIIAV IRSTB051). The study complied with the provisions of the Good Clinical Practice guidelines and the Declaration of Helsinki and local laws and fulfilled Regulation (EU) 2016/679 of the European Parliament and the Council of 27 April 2016 on the protection of natural persons with regard to the processing of personal data.

### 4.2. Sample Collection, PBMC Preparation and Cytometry Data Collection

Peripheral blood mononuclear cells (PBMC) were separated by density gradient centrifugation using Lymphosep (Biowest, Riverside, MO, USA). Then, fresh PBMC were fluorescently stained with specific antibodies and analyzed by flow cytometry.

Flow cytometric analysis was performed using a FACSCanto flow cytometer (Becton–Dickinson, Franklin Lakes, NJ, USA) equipped with 488 nm (blue) and 633 (red) lasers. For each sample, 100,000 events were recorded. Acquisition and analysis gates were set on forward (FSC) and side scatter (SSC) properties of cells. T cell subsets were separated according to CD8 expression into non-cytotoxic T cells (CD3^+^CD8^−^) and cytotoxic T cells (CD3^+^CD8^+^), whereas NK and NK/T cells were according to CD56 with or without CD3/CD8 expression. CD3, CD8 and CD56 antibodies were from Miltenyi Biotec (Bergisch Gladbach, Germany). Monocytes were identified according to side scatter and CD14 profiles. CD14^+^ cells were subsequently separated according to CD16 expression into classical (CD14^++^), intermediate (CD14^++^CD16^+^) and nonclassical (CD16^++^) subsets.

The analysis of MDSC was performed until 4 h from sample collection. The following antibodies were used for the analysis of MO-MDSC: CD14, CD11b, CD33 and HLA-DR (all from Miltenyi Biotec). Appropriate isotype controls were included for each sample. Flow cytometry data were analyzed using FacsDiva software v6.1.3 (Becton Dickinson, Franklin Lakes, NJ, USA).

### 4.3. Statistical Analysis

Categorical variables were compared with Fisher’s exact test. OS was defined as the time interval from the first day of treatment to the day of death or last follow-up visit. PFS was defined as the time interval from the first day of treatment to the day of tumor progression or death, whichever occurred first. OS and PFS were estimated by the Kaplan–Meier method.

MedCalc package (MedCalc^®^ version 16.8.4; MedCalc Software Ltd., Ostend, Belgium) was used for statistical analysis.

## 5. Conclusions

Our results, although obtained in a small sample size and in a retrospective analysis, shed light on some features of IT activity in HCC. Analysis of patterns of response highlighted a heterogeneous response of different organ lesions to nivolumab, with a greater sensitivity of lung metastases. On the other hand, PBMC analysis showed peculiar alterations, both at baseline and during treatment with nivolumab, in a case of HPD, and identified features with a possible prognostic role.

These results are worthy of further investigation and confirmation, possibly in a prospective study, since a deeper understanding of mechanisms underlying IT activity in HCC could result in a more appropriate treatment strategy, taking advantage of the combination of several therapeutic approaches.

## Figures and Tables

**Table 1:** Patient baseline characteristics.

	*n*	%
**Age**		
Median 66 (range 56–82)		
≤64 years	2	20
65–69 years	5	50
≥70 years	3	30
**Sex**		
Male	8	80
Female	2	20
**ECOG PS**		
0	6	60
1	4	40
**Cause of HCC** ^a^		
HBV	2	
HCV	3	
alcohol	4	
metabolic syndrome	1	
unknown	1	
**Site of lesions**		
Liver	10	100
Extrahepatic disease ^b^	8	80
Lung	4	
Peritoneum	5	
Bone	3	
Lymph nodes	1	
Soft tissues	1	
**Child–Pugh score**		
5	4	40
6	6	60
≥7	0	0
α-fetoprotein ≥ 400 ng/mL	1	10
**Previous treatments**		
Locoregional therapy ^c^	9	90
Surgery	7	
Thermal ablation	5	
TACE	6	
Systemic therapy	10	100
Sorafenib	10	100
Reason for discontinuation of sorafenib		
Progressive disease	9	90
Toxicity	1	10

^a^ One patient had coexisting HCV and alcoholic etiologies. ^b^ Some patients had more than one extrahepatic site involved. ^c^ Some patients had received more than one locoregional therapy. ECOG PS, Eastern Cooperative Oncology Group Performance Status; TACE, transarterial chemoembolization.

**Table 2:** Treatment outcomes.

Patient	Sex	Age	Site of Lesions	Survival (Months)	Time to Progression (Months)	Best Response	Reason for Discontinuation	Adverse Effects	Subsequent Therapy
1 ^#^	M	74	liver	23.6 *	NR	SD	NA (ongoing)	-	NA
2 ^#^	F	82	liver	17.7	6.2	SD	PD	hypothyroidism (G2)	capecitabine
3 ^#^	F	56	liver, peritoneum, lymph nodes, bone	2.7	2.2	PD	PD	pneumonia (G3)	-
4 ^#^	M	66	liver, bone	8.4	4.4	SD	PD	skin (G1)	-
5	M	57	liver, lung, peritoneum, bone	29.1 *	NR	PR	NA (ongoing)	-	NA
6	M	66	liver, lung	5.7	3.1	PD	PD	-	-
7	M	74	liver, lung	9.5	8.3	SD	PD	pneumonia (G1)	-
8	M	69	liver, peritoneum	16.3	9.6	SD	PD	-	clinical trial
9	M	65	liver, peritoneum, soft tissues	15.5 *	NR	SD	NA (ongoing)	-	NA
10	M	66	liver, lung, peritoneum	§	1.7	PD	COVID-19	-	§

^#^ Patient with peripheral blood mononuclear cell (PBMC) collection. * Partial data (patient alive). § Lost to follow-up. M, male; F, female; NR, progression not reached; NA, not applicable; PR, partial response; SD, stable disease; PD, progressive disease; G, grade.

## Data Availability

Data available on request.

## Supplementary Materials

The following are available online at https://www.mdpi.com/2072-6694/13/2/213/s1, Figure S1: Gating strategy and representative dot plots of flow cytometry analysis of PBMC; Figure S2: Analysis of monocytes by flow cytometry; Figure S3: Analysis of monocytes by standard blood count examination.
