# Supplementary material for: Heterogeneity of Response and Immune System Activity during Treatment with Nivolumab in Hepatocellular Carcinoma: Results from a Single-Institution Retrospective Analysis
Source: Cancers (Basel). 2021 Jan 8;13(2):213. doi: 10.3390/cancers13020213 (PMC7827490; doi:10.3390/cancers13020213)
Supplement: Supplementary file 1 [file cancers-13-00213-s001.pdf]

cancers

Supplementary Materials: Heterogeneity of Response and Immune System Activity during Treatment with Nivolumab in Hepatocellular Carcinoma: Results from a Single-Institution Retrospective Analysis

Ilario Giovanni Rapposelli, Serena De Matteis, Paola Lanuti, Martina Valgiusti, Giulia Bartolini, Paola Ulivi, Giorgia Marisi, Federica Pedica, Valentina Burgio, Giovanni Luca Frassineti, Stefano Cascinu and Andrea Casadei-Gardini

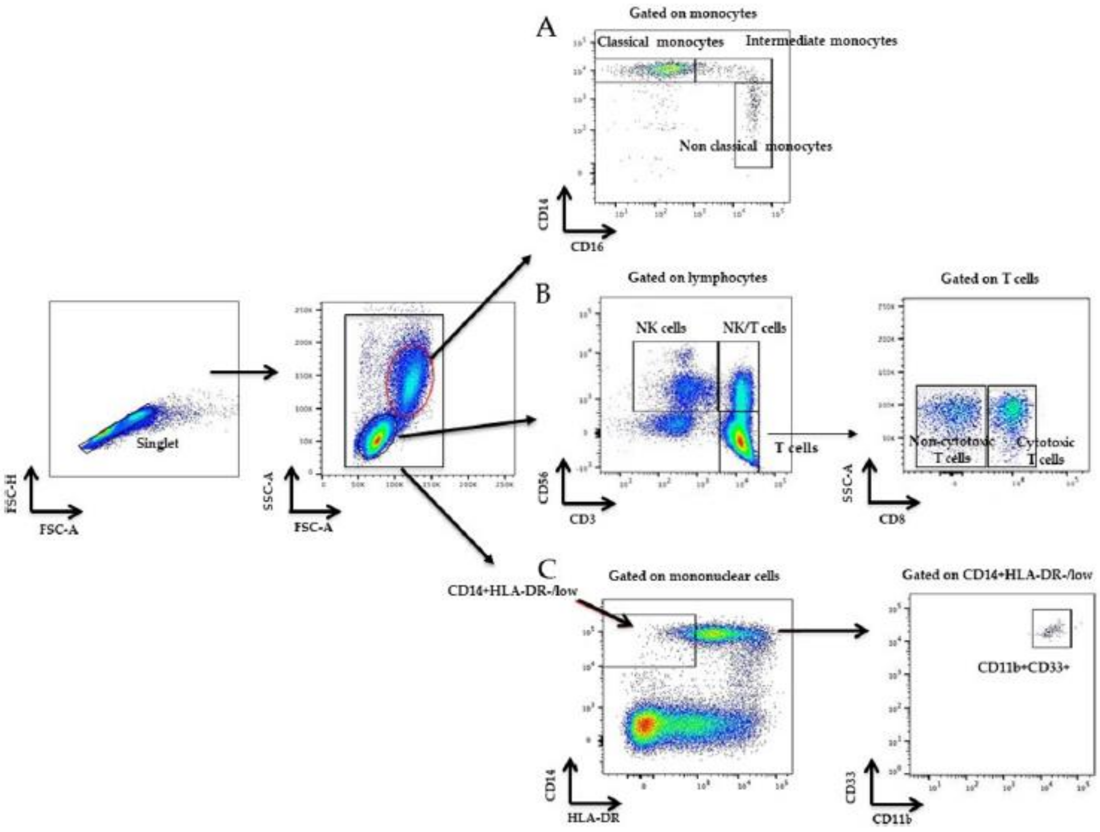

Figure S1. Gating strategy and representative dot plots of flow cytometry analysis of PBMC. After exclusion of doublets, (**A**) monocytic subpopulations were identified according to CD14 and CD16 expression into classical (CD14++), intermediate (CD14++CD16+) and non-classical (CD16++) subsets. (**B**) T cell subsets were separated according to CD3 and CD56 expression into NK (CD3-CD56+), NK/T (CD3+CD56+) and T (CD3+CD56-) cells. Then, T cell population was gated, and cytotoxic and non-cytotoxic T cells were identified according to side scatter and CD8 profile. (**C**) CD14+HLA-DR-/low cell subsets were gated and the proportion of CD11b+CD33+ was evaluated to identify MO-MDSC.

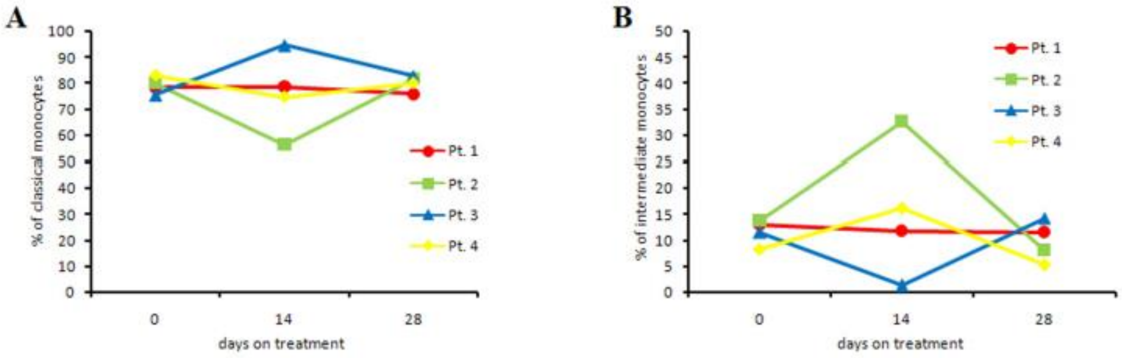

Figure S2. Analysis of monocytes by flow cytometry**.** (**A**) Percentage of classical monocytes over the courseof therapy with nivolumab (at baseline, after 14 and after 28 days). (**B**) Percentage of intermediate monocytes over the course of therapy with nivolumab (at baseline, after 14 and after 28 days). Analysis has been performed in the first 4 patients. Numbering of patients in the two panels is the same reported in Table 2.

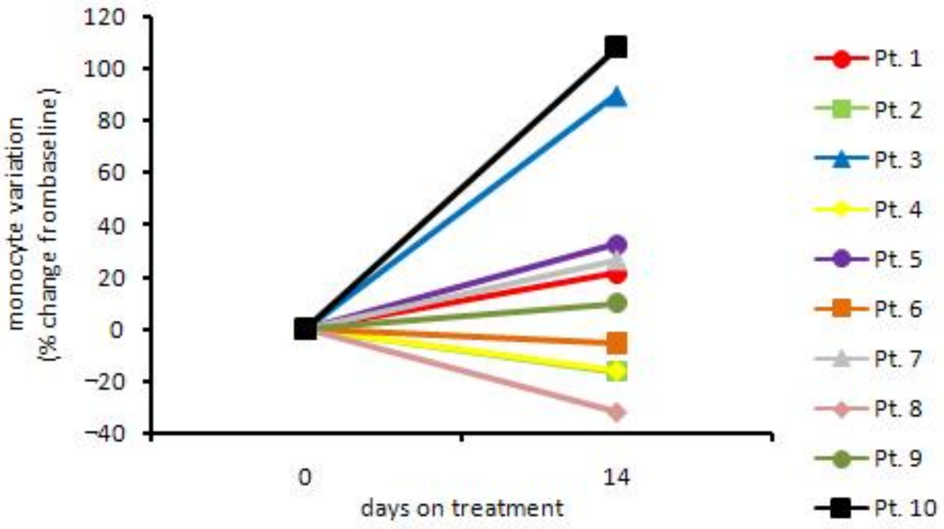

Figure S3. Analysis of monocytes by standard blood count examination. Results of monocyte count at baseline and after 14 days of treatment with nivolumab are reported and depicted as percentage change from baseline. Analysis has been performed in all 10 patients. Numbering of patients in the two panels is the same reported in Table 2.
